# Supplementary material for: Evaluating vector competence for Yellow fever in the Caribbean
Source: Nat Commun. 2024 Feb 9;15:1236. doi: 10.1038/s41467-024-45116-2 (PMC10858021; doi:10.1038/s41467-024-45116-2)

Evaluating vector competence for Yellow fever in the Caribbean

Gaelle Gabiane,[1,2] Chloé Bohers,[1] Laurence Mousson,[1] Thomas Obadia,[3,4] Rhoel R. Dinglasan,[5] Marie Vazeille,[1] Catherine Dauga,[1] Marine Viglietta,[1] André Yébakima,[6] Anubis Vega-Rúa,[7] Gladys Gutiérrez Bugallo,[7,8] Rosa Margarita Gélvez Ramírez,[9,10] Fabrice Sonor,[11,12] Manuel Etienne,[11] Nathalie Duclovel-Pame,[12] Alain Blateau,[12] Juliette Smith-Ravin,[13] Xavier De Lamballerie,[10] Anna-Bella Failloux[1*]

[1]Institut Pasteur, Université Paris Cité, Arboviruses and Insect Vectors, Paris, France
[2]Université des Antilles, Ecole Doctorale 589, Schœlcher, Martinique
[3]Institut Pasteur, Université Paris Cité, Bioinformatics and Biostatistics Hub, France
[4]Institut Pasteur, Université Paris Cité, G5 Infectious Disease Epidemiology and Analytics, Paris, France
[5]University of Florida, Department of Infectious Diseases & Immunology and Emerging Pathogens Institute, College of Veterinary Medicine, Gainesville, Florida, USA
[6]VECCOTRA, Rivière Salée, Martinique
[7]Institut Pasteur de Guadeloupe, Laboratory of Vector Control Research, Unit Transmission Reservoir and Pathogens Diversity, Les Abymes, Guadeloupe
[8]Department of Vector Control, Center for Research, Diagnostic, and Reference, Institute of Tropical Medicine Pedro Kouri, Havana, Cuba.
[9]Centro de Atención y Diagnóstico de Enfermedades Infecciosas, Fundación INFOVIDA, Bucaramanga, Colombia
[10]Unité des Virus Emergents (UVE), Aix Marseille Université, IRD 190, Inserm 1207, IHU Méditerranée Infection, Marseille, France
[11]Centre de Démoustication et de Recherches Entomologiques, Lutte antivectorielle, Martinique
[12]Agence Régionale de Santé, Direction de la Santé Publique, Martinique
[13]Groupe de recherche Biospheres Université des Antilles, Campus de Schœlcher, Martinique

[*]Corresponding author. Email: anna-bella.failloux@pasteur.fr

Table S1. Populations of *Aedes aegypti* from Martinique and neighboring island and continental countries collected in 2019-2020.

Population name	Collection		Generation used for infection
	Island or country	Site	
**Martinique**			
Anse Maroquet	Martinique	Le Vauclin	F4
Bois lézard	Martinique	Gros Morne	F6
Cap Chevalier	Martinique	Sainte-Anne	F4
Diamant	Martinique	Le Diamant	F3
Lareinty	Martinique	Le Lamentin	F5
Macouba	Martinique	Macouba	F4
Morne-Rouge	Martinique	Le Morne-Rouge	F2
Sainte-Thérèse	Martinique	Fort-de-France	F4
Tartane	Martinique	La Trinité	F4
**Caribbean islands**			
Les Abymes	Guadeloupe	Les Abymes	F2
CTU	Haïti	Port-au-Prince	F3
Papin	Haïti	Port-à-Piment	F2
Havana	Cuba	La Habana	F2
**Continent**			
Nor-Oriental	Colombia	Santa Cruz	F2
Gainesville	United States	Gainesville	F3

Table S2. Infection (IR), stepwise dissemination (SDR), stepwise transmission (STR) rates, and viral loads (midgut, carcass, saliva) of nine *Aedes aegypti* populations from Martinique examined 14 days after exposure to an infectious blood meal containing one YFV strain (Bolivia, Ghana, Nigeria, Sudan or Uganda). Mosquitoes were exposed to an infectious blood meal at a titer of 10^7 FFU/mL using an Hemotek system maintained at 37 °C. Engorged mosquitoes were kept for 14 days in controlled conditions and each surviving mosquito was dissected to isolate the midgut and the carcass, and collect saliva to estimate the viral load by titration. IR, proportion of mosquitoes with an infected midgut among mosquitoes exposed to the blood meal; SDR, proportion of mosquitoes with an infected carcass among mosquitoes with an infected midgut; STR, proportion of mosquitoes with infectious saliva among mosquitoes with an infected carcass. In brackets, number of tested mosquitoes.

Virus	Indice	Anses Maroquet	Bois lézard	Cap Chevalier	Diamant	Lareinty	Macouba	Morne-Rouge	Sainte-Thérèse	Tartane
YFV Bolivia	IR	62.5 (32)	90.5 (21)	65.6 (32)	75.0 (32)	59.4 (32)	93.8 (32)	53.1 (32)	81.3 (32)	43.8 (32)
	SDR	90.0 (20)	63.2 (19)	76.2 (21)	70.8 (24)	73.7 (19)	70.0 (30)	52.9 (17)	19.2 (26)	78.6 (14)
	STR	0 (18)	0 (12)	6.3 (16)	11.8 (17)	14.3 (14)	9.5 (21)	0 (10)	20.0(5)	9.1 (11)
	Viral load in midgut	43086 (20)	12983 (19)	9779 (21)	13578 (24)	12162 (19)	23328 (30)	10617 (17)	8434 (26)	25098 (14)
	Viral load in carcass	45378 (18)	5458 (12)	6093 (16)	6737 (17)	4638 (14)	30298 (21)	787 (10)	11412 (5)	31832 (11)
	Viral load in saliva	- (0)	- (0)	2 (1)	9 (2)	19 (2)	4 (2)	- (0)	112 (1)	2 (1)
YFV Ghana	IR	6.3 (32)	3.0 (33)	37.5 (32)	18.8 (32)	25.0 (32)	37.5 (32)	6.3 (32)	43.8 (32)	37.5 (32)
	SDR	0 (2)	100 (1)	33.3 (12)	16.7 (6)	62.5 (8)	58.3 (12)	50.0 (2)	35.7 (14)	25.0 (12)
	STR	- (0)	0 (2)	0 (5)	0 (4)	0 (6)	0 (7)	0 (1)	0 (6)	0 (3)
	Viral load in midgut	18 (2)	600 (1)	5673 (12)	482 (6)	1350 (8)	725 (12)	192 (2)	424 (14)	874 (12)
	Viral load in carcass	- (0)	24 (2)	932 (5)	45 (4)	1452 (6)	119 (7)	24 (1)	62 (6)	72 (3)
	Viral load in saliva	- (0)	- (0)	- (0)	- (0)	- (0)	- (0)	- (0)	- (0)	- (0)
YFV Nigeria	IR	45.8 (24)	12.5 (32)	87.0 (23)	71.9 (32)	91.7 (24)	93.8 (32)	53.1 (32)	59.4 (32)	66.7 (24)
	SDR	45.5 (11)	0 (4)	90.0 (20)	69.6 (23)	31.8 (22)	76.7 (30)	58.8 (17)	42.1 (19)	18.8 (16)
	STR	20.0 (5)	- (0)	38.9 (18)	2.05 (16)	14.3 (7)	8.7 (23)	10.0 (10)	0 (8)	0 (3)
	Viral load in midgut	557 (11)	24 (4)	7731 (20)	2298 (23)	1605 (22)	13924 (30)	1858 (17)	232 (19)	4624 (16)
	Viral load in carcass	419 (5)	- (0)	4404 (18)	1992 (16)	245 (7)	4535 (23)	1289 (10)	249 (8)	20 (3)
	Viral load in saliva	2 (1)	- (0)	3 (7)	11 (5)	2 (1)	61 (2)	8 (1)	- (0)	6 (1)
YFV Sudan	IR	41.7 (24)	37.5 (24)	72.7 (33)	78.1 (32)	54.2 (24)	90.6 (32)	84.4 (32)	31.3 (32)	95.8 (24)
	SDR	30 (10)	66.7 (9)	70.8 (24)	68.0 (25)	61.5 (13)	75.9 (29)	48.1 (27)	0 (10)	52.2 (23)
	STR	0 (3)	0 (6)	0 (18)	5.6 (18)	37.5 (8)	18.2 (22)	0 (13)	- (0)	0 (12)
	Viral load in midgut	2163 (10)	372 (9)	1802 (24)	827 (25)	266 (13)	1604 (29)	1838 (27)	75 (10)	4740 (23)
	Viral load in carcass	1268 (3)	224 (6)	1052 (18)	459 (18)	450 (8)	944 (22)	7982 (13)	- (0)	182 (12)
	Viral load in saliva	- (0)	- (0)	- (0)	2 (1)	376 (3)	29 (4)	- (0)	- (0)	- (0)
YFV Uganda	IR	79.2 (24)	75 (24)	96.9 (32)	87.5 (32)	84.4 (32)	100 (32)	50 (32)	96.9 (32)	87.0 (23)
	SDR	94.7 (19)	55.6 (18)	100 (31)	78.6 (28)	74.1 (27)	93.8 (32)	81.3 (16)	32.3 (31)	50.0 (20)
	STR	22.2 (18)	50.0 (10)	32.3 (31)	0 (22)	45.0 (20)	16.7 (30)	15.4 (13)	0 (10)	30.0 (10)
	Viral load in midgut	2102 (19)	2393 (18)	14030 (31)	7581 (28)	5544 (27)	15435 (32)	8066 (16)	552 (31)	17832 (20)
	Viral load in carcass	1819 (18)	1430 (10)	13709 (31)	1843 (22)	41101 (20)	11117 (30)	6823 (13)	102 (10)	103 (10)
	Viral load in saliva	4 (4)	221 (7)	23 (10)	- (0)	342 (10)	32 (5)	73 (2)	- (0)	28 (3)

Table S3. Infection (IR), stepwise dissemination (SDR), stepwise transmission (STR) rates, and viral loads (midgut, carcass, saliva) of nine *Aedes aegypti* populations from Martinique examined 21 days after exposure to an infectious blood meal containing one YFV strain (Bolivia, Ghana, Nigeria, Sudan or Uganda). Mosquitoes were exposed to an infectious blood meal at a titer of 10^7 FFU/mL using an Hemotek system maintained at 37 °C. Engorged mosquitoes were kept for 21 days in controlled conditions and each surviving mosquito was dissected to isolate the midgut and the carcass, and to collect saliva to estimate the viral load by titration. IR, proportion of mosquitoes with an infected midgut among mosquitoes exposed to the blood meal; SDR, proportion of mosquitoes with an infected carcass among mosquitoes with an infected midgut; STR, proportion of mosquitoes with infectious saliva among mosquitoes with an infected carcass. In brackets, number of tested mosquitoes.

Virus	Indice	Anses Maroquet	Bois lézard	Cap Chevalier	Diamant	Lareinty	Macouba	Morne-Rouge	Sainte-Thérèse	Tartane
YFV Bolivia	IR	59.4 (32)	77.3 (22)	53.1 (32)	82.5 (40)	65.6 (32)	81.3 (32)	65.6 (32)	90 (40)	35.3 (34)
	SDR	78.9 (19)	82.4 (17)	82.4 (17)	81.8 (33)	71.4 (21)	76.9 (26)	81.0 (21)	66.7 (36)	83.3 (12)
	STR	20.0 (15)	14.3 (14)	14.3 (14)	11.1 (27)	6.7 (15)	25.0 (20)	17.6 (17)	4.2 (24)	10.0 (10)
	Viral load in midgut	40745 (19)	5558 (17)	17505 (17)	8899 (33)	14328 (21)	6004 (26)	17471 (21)	20424 (36)	27047 (12)
	Viral load in carcass	199921 (15)	25718 (14)	89550 (14)	23887 (27)	41626 (15)	15507 (20)	25953 (17)	68183 (24)	118344 (10)
	Viral load in saliva	97 (3)	140 (2)	26 (2)	102 (3)	49 (1)	130 (5)	95 (3)	40 (1)	320 (1)
YFV Ghana	IR	35.0 (20)	3.1 (32)	25.0 (32)	37.5 (32)	28.1 (32)	33.3 (33)	18.8 (32)	51.9 (27)	37.5 (32)
	SDR	71.4 (7)	0 (1)	37.5 (8)	8.3 (12)	44.4 (9)	45.5 (11)	16.7 (6)	0 (14)	25.0 (12)
	STR	0 (7)	- (0)	0 (3)	33.3 (3)	25.0 (4)	0 (8)	0 (2)	- (0)	0 (3)
	Viral load in midgut	15108 (7)	84 (1)	2281 (8)	838 (12)	3376 (9)	1006 (11)	1021 (6)	194 (14)	782 (12)
	Viral load in carcass	1126 (7)	- (0)	2900 (3)	720 (3)	516 (4)	286 (8)	162 (2)	- (0)	32 (3)
	Viral load in saliva	- (0)	- (0)	- (0)	2 (1)	2 (1)	- (0)	- (0)	- (0)	- (0)
YFV Nigeria	IR	66.7 (15)	58.3 (24)	76.5 (17)	71.9 (32)	83.3 (24)	84.8 (33)	31.3 (32)	68.8 (32)	62.5 (24)
	SDR	50.0 (10)	57.1 (14)	100 (13)	69.6 (23)	45.0 (20)	85.7 (28)	90.0 (10)	36.4 (22)	80.0 (15)
	STR	0 (5)	37.5 (8)	7.1 (14)	29.4 (17)	11.1 (9)	29.2 (24)	33.3 (9)	25.0 (8)	25.0 (12)
	Viral load in midgut	3011 (10)	1432 (14)	2903 (13)	2128 (23)	8692 (20)	7953 (28)	1512 (10)	1051 (22)	12746 (15)
	Viral load in carcass	295 (5)	23976 (8)	2696 (14)	7497 (17)	1345 (9)	35667 (24)	9952 (9)	372 (8)	4431 (12)
	Viral load in saliva	440 (1)	17 (3)	3 (2)	24 (6)	320 (1)	4 (8)	4 (3)	18 (2)	24 (3)
YFV Sudan	IR	29.2 (24)	29.2 (24)	64.0 (25)	81.3 (32)	58.3 (24)	97.1 (35)	80.6 (31)	53.1 (32)	91.7 (24)
	SDR	14.3 (7)	100 (7)	93.8 (16)	88.5 (26)	42.5 (14)	91.2 (34)	72.0 (25)	35.3 (17)	36.4 (22)
	STR	100 (1)	57.1 (7)	18.8 (16)	17.4 (23)	50.0 (6)	16.1 (31)	31.6 (19)	42.9 (7)	12.5 (8)
	Viral load in midgut	245 (7)	157 (7)	1315 (16)	2055 (26)	405 (14)	1633 (34)	2352 (25)	264 (17)	4092 (22)
	Viral load in carcass	36 (1)	131828 (7)	11786 (16)	2411 (23)	890 (6)	41985 (31)	49179 (19)	420 (7)	1500 (8)
	Viral load in saliva	17 (1)	31 (4)	2 (3)	43 (4)	1626 (4)	209 (5)	52 (7)	125 (6)	440 (1)
YFV Uganda	IR	77.8 (18)	66.7 (24)	86.7 (30)	90.6 (32)	87.5 (24)	100 (33)	71.4 (28)	96.7 (30)	95.8 (24)
	SDR	64.3 (14)	100 (16)	100 (26)	82.8 (29)	95.2 (21)	97.0 (33)	95.0 (20)	79.3 (29)	82.6 (23)
	STR	44.4 (9)	50.0 (16)	26.9 (26)	37.5 (24)	45.0 (20)	31.3 (32)	15.0 (20)	20.8 (24)	42.1 (19)
	Viral load in midgut	3317 (14)	2016 (16)	4771 (26)	9318 (29)	3363 (21)	10327 (33)	2221 (20)	2319 (29)	10674 (23)
	Viral load in carcass	136 (9)	92400 (16)	77676 (26)	45550 (24)	21751 (20)	34178 (32)	9795 (20)	6442 (24)	8691 (19)
	Viral load in saliva	32 (5)	1359 (8)	10 (7)	35 (9)	118 (9)	698 (10)	39 (3)	18 (5)	404 (8)

Table S4. Infection (IR), stepwise dissemination (SDR), stepwise transmission (STR) rates, and viral loads (midgut, carcass, saliva) of six *Aedes aegypti* populations from the Caribbean-Americas region examined 21 days after exposure to an infectious blood meal containing one YFV strain (Bolivia, Ghana, Nigeria, Sudan or Uganda). Mosquitoes were exposed to an infectious blood meal at a titer of 10^7 FFU/mL using an Hemotek system maintained at 37 °C. Engorged mosquitoes were kept for 21 days in controlled conditions and each surviving mosquito was dissected to isolate the midgut and the carcass and to collect saliva to estimate the viral load by titration. IR, proportion of mosquitoes with an infected midgut among mosquitoes exposed to the blood meal; SDR, proportion of mosquitoes with an infected carcass among mosquitoes with an infected midgut; STR, proportion of mosquitoes with infectious saliva among mosquitoes with an infected carcass. In brackets, number of tested mosquitoes.

Virus	Indice	Abymes	CTU	Papin	La Havane	Gainesville	Nor-Oriental
YFV Bolivia	IR	93.8 (32)	37.5 (48)	47.7 (44)	50 (24)	67.5 (40)	45.8 (24)
	SDR	83.3 (30)	50.0 (18)	66.7 (21)	50.0 (12)	66.7 (27)	45.5 (11)
	STR	12.0 (25)	22.2 (9)	35.7 (14)	33.3 (6)	27.8 (18)	20.0 (5)
	Viral load in midgut	18297 (30)	8140 (18)	5066 (21)	3356 (12)	8539 (27)	2650 (11)
	Viral load in carcass	50976 (25)	5876 (9)	37389 (14)	94200 (6)	42805 (18)	48249 (5)
	Viral load in saliva	100 (3)	8 (2)	19 (5)	24 (2)	59 (5)	12 (1)
YFV Ghana	IR	25.0 (24)	25.0 (32)	25.0 (32)	41.7 (24)	11.5 (26)	16.7 (24)
	SDR	16.7 (6)	25.0 (8)	50.0 (8)	50.0 (10)	33.3 (3)	50.0 (4)
	STR	0 (1)	0 (2)	25.0 (4)	0 (5)	0 (1)	0 (2)
	Viral load in midgut	576 (6)	72 (8)	142 (8)	870 (10)	888 (3)	1332 (4)
	Viral load in carcass	480 (1)	366 (2)	222 (4)	885 (5)	36 (1)	2568 (2)
	Viral load in saliva	- (0)	- (0)	14 (1)	- (0)	- (0)	- (0)
YFV Nigeria	IR	79.2 (24)	62.5 (24)	78.1 (32)	75.0 (24)	92.6 (27)	46.7 (15)
	SDR	42.1 (19)	6.7 (15)	56.0 (25)	77.8 (18)	40.0 (25)	57.1 (7)
	STR	25.0 (8)	0 (1)	6.7 (15)	26.7 (15)	0 (10)	25.0 (4)
	Viral load in midgut	3392 (19)	8104 (15)	1507 (25)	1277 (18)	891 (25)	3084 (7)
	Viral load in carcass	372 (8)	480 (1)	57465 (15)	13745 (15)	7140 (10)	2706 (4)
	Viral load in saliva	60 (2)	- (0)	2 (1)	64 (4)	- (0)	8 (1)
YFV Sudan	IR	95.8 (24)	66.7 (24)	84.4 (32)	33.3 (24)	66.7 (27)	52.2 (23)
	SDR	69.6 (23)	43.8 (16)	63.0 (27)	75.0 (8)	50.0 (18)	33.3 (12)
	STR	12.5 (16)	0 (7)	5.9 (17)	16.7 (6)	0 (10)	0 (4)
	Viral load in midgut	1810 (23)	3871 (16)	2001 (27)	525 (8)	847 (18)	1004 (12)
	Viral load in carcass	804 (16)	3598 (7)	22396 (17)	13900 (6)	2616 (10)	6030 (4)
	Viral load in saliva	78 (2)	- (0)	200 (1)	6 (1)	- (0)	- (0)
YFV Uganda	IR	66.7 (24)	95.8 (24)	84.4 (32)	100 (24)	93.3 (30)	50.0 (24)
	SDR	68.8 (16)	82.6 (23)	77.8 (27)	100 (24)	53.6 (28)	100 (12)
	STR	18.2 (11)	31.6 (19)	23.8 (21)	20.8 (24)	6.7 (15)	8.3 (12)
	Viral load in midgut	745 (16)	14086 (23)	6661 (27)	3009 (24)	315 (28)	10948 (12)
	Viral load in carcass	540 (11)	4441 (19)	47700 (21)	119708 (24)	1573 (15)	2699 (12)
	Viral load in saliva	78 (2)	216 (6)	3808 (5)	68 (5)	3 (1)	2 (1)

Figure S1. Phylogeny of YFV genotypes based on complete genomes. A multiple-sequence alignment was performed using muscle implemented in Seaview 5.04 (Gouy et al. Seaview version 4: A multiplatform graphical user interface for sequence alignment and phylogenetic tree building. Mol Biol Evol 2010; 27(2):221-4). A phylogenetic tree was inferred on the whole genome using the maximum-likelihood (ML) method, using the best substitution model (GTR + Gamma). The robustness of each node was assessed by bootstrap resampling (100 replicates). YFV Bolivia (GenBank accession number: MF004382), Nigeria (AF369681), Ghana (MF405338), Uganda (https://www.european-virus-archive.com/virus/yellow-fever-virus-strain-uveyfv1948ugmr896-tvp3236), Sudan (MF004383), and Angola (AY968064.1).

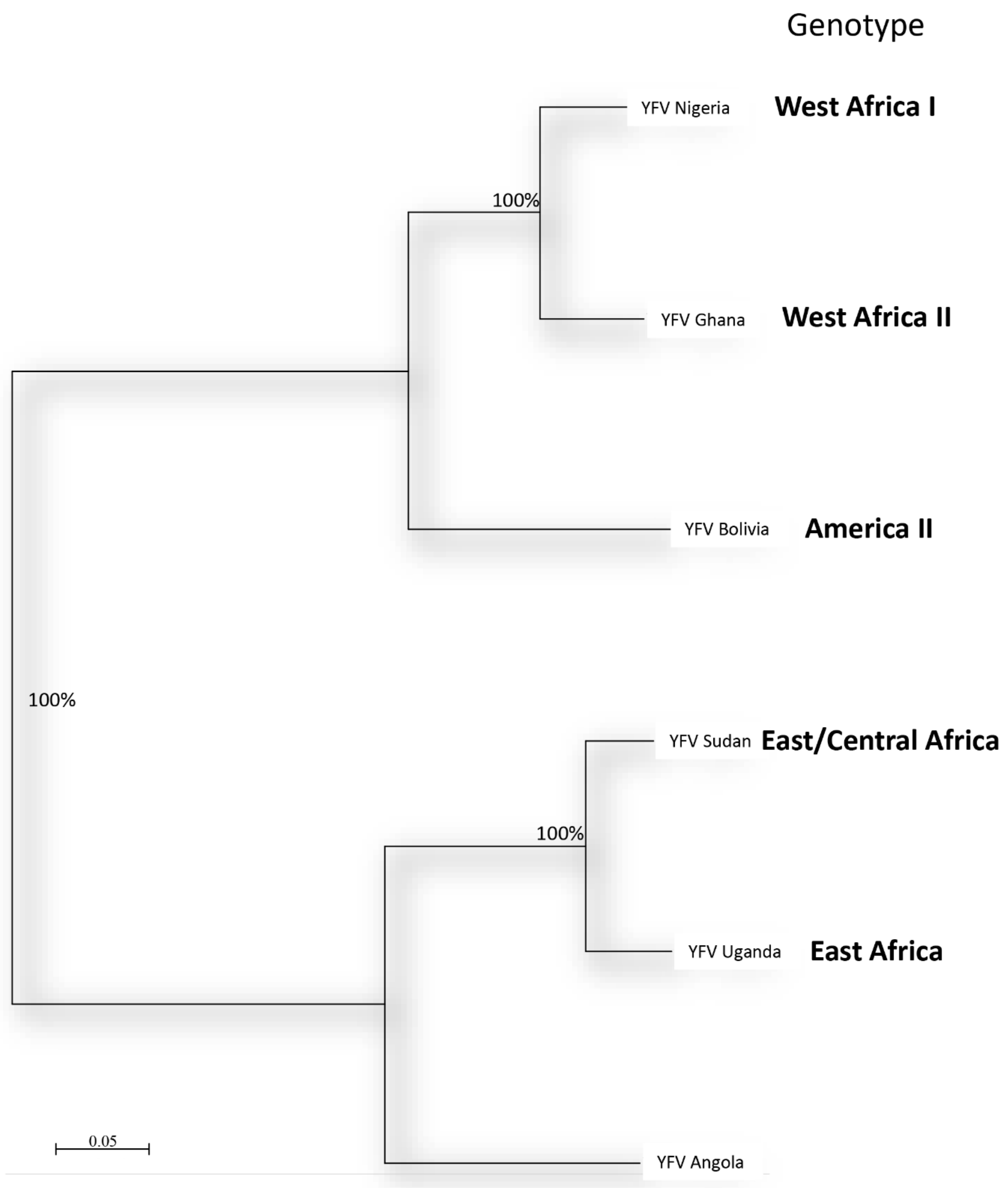

Figure S2. Experimental design of mosquito infections for vector competence analysis (infection, dissemination, and transmission). IR, infection rate; SDR, stepwise dissemination rate; STR, stepwise transmission rate. Created with BioRender.com.

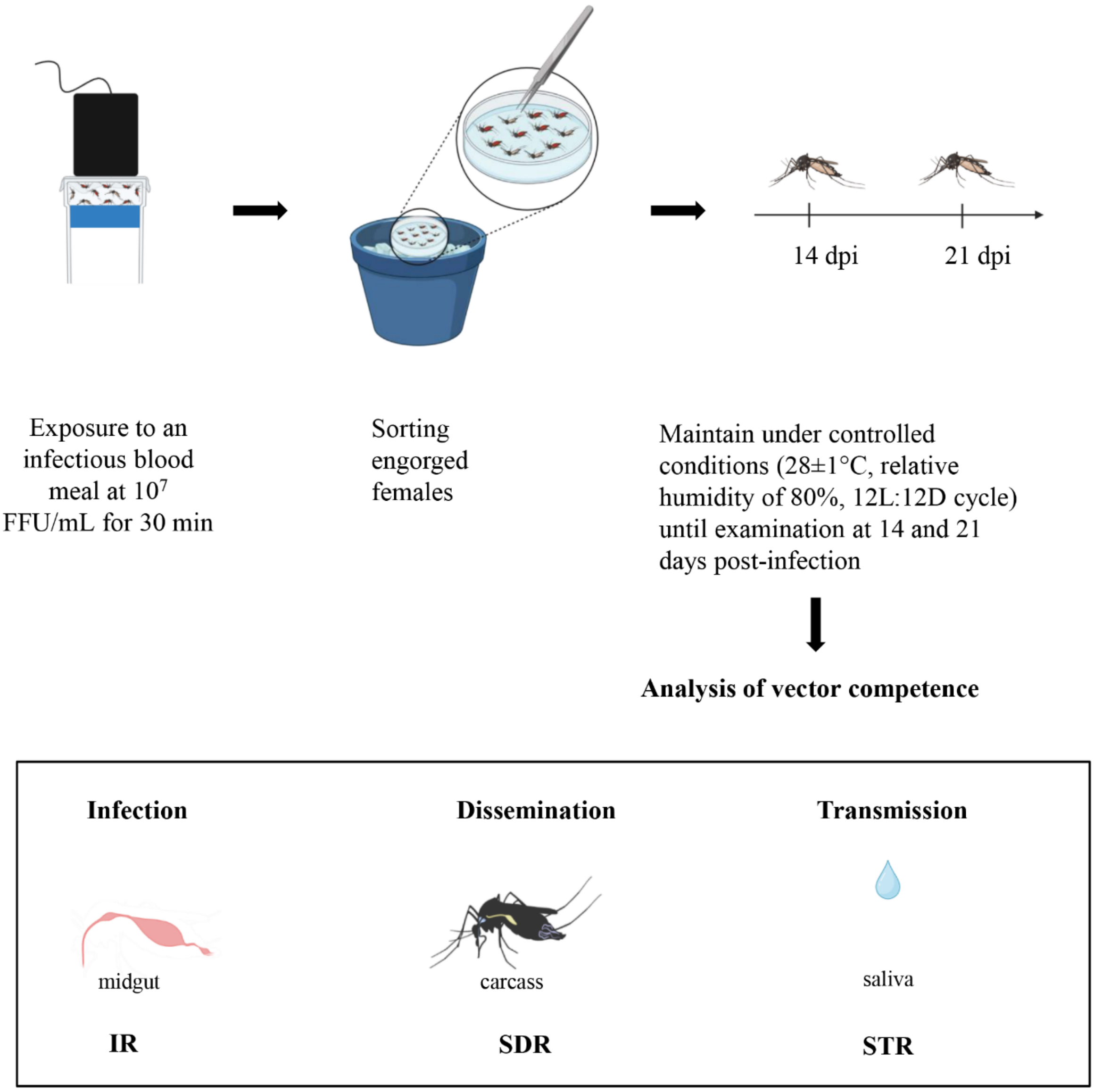

Figure S3. Infection, stepwise dissemination, and stepwise transmission rates of *Aedes aegypti* Tartane (Martinique) examined 7, 14, 21, and 28 days after an infectious meal containing YFV Bolivia, provided at two titers, 10^5 and 10^7 FFU/mL. The error bars correspond to the confidence intervals (95%) for IR (A, D), SDR (B, E), and STR (C, F); ** $0.001 \leq p < 0.01$ ($p=0.002$ for E) by Fisher's exact test (two-sided). In brackets are the numbers of mosquitoes tested. Source data are provided as a Source Data file.

As viral infection, dissemination, and transmission were only detected when mosquitoes were exposed to a blood meal containing 10^7 FFU/mL, this blood meal titer was selected for assessing the vector competence of all mosquito populations.

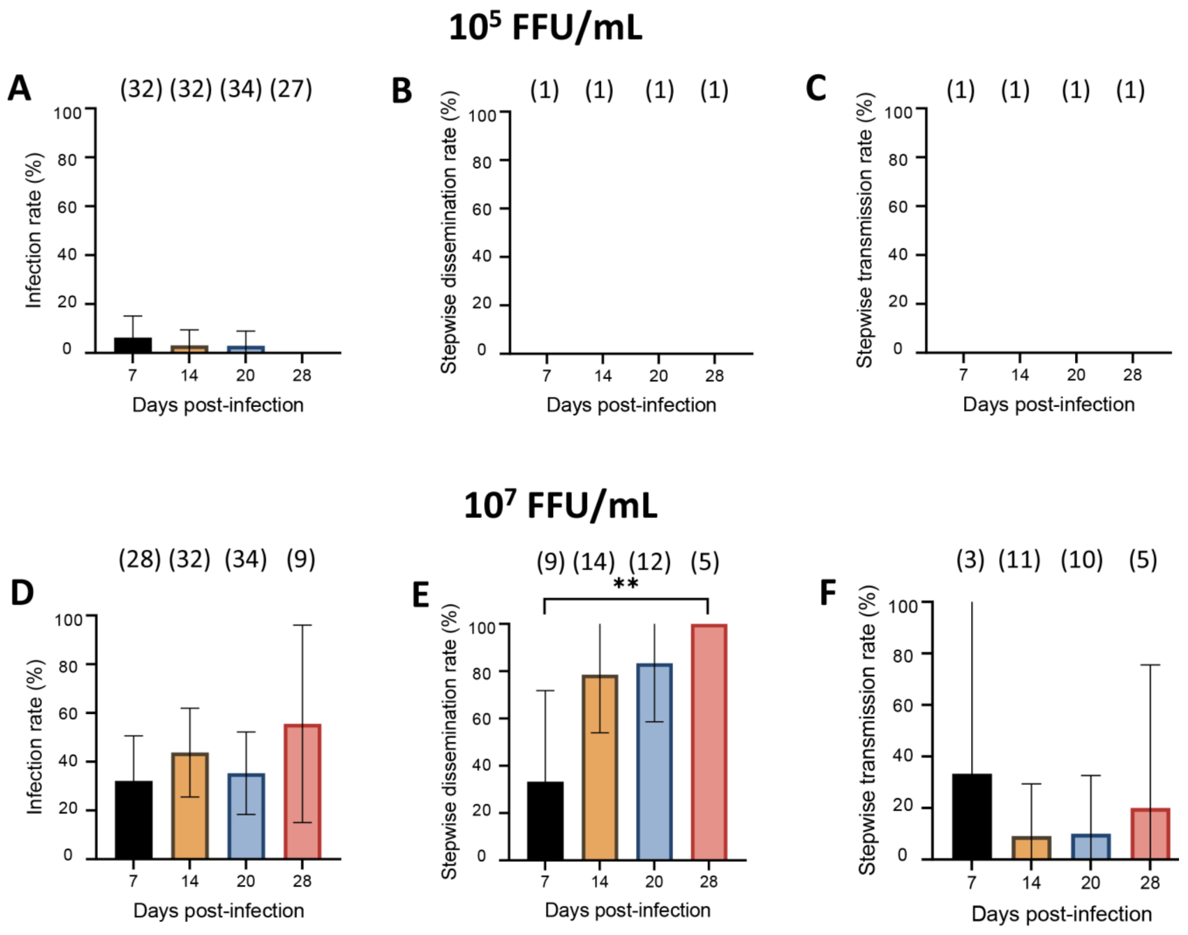

Figure S4. Infection rates (A-E) and viral loads in mosquito midgut (F-J) of nine *Aedes aegypti* populations from Martinique examined 14 days after exposure to an infectious blood meal containing one YFV strain (Bolivia, Ghana, Nigeria, Sudan or Uganda). Mosquitoes were exposed to an infectious blood meal at a titer of 10^7 FFU/mL using an Hemotek system maintained at 37 °C. Engorged mosquitoes were kept for 14 days in controlled conditions and then dissected to isolate the midgut for estimating the viral load by titration. (A-E) the infection rate was defined as the proportion of mosquitoes with an infected midgut among the total number of mosquitoes exposed to the blood meal. The error bars correspond to the confidence intervals (95%) for IR (Table S2); ****$p<0.0001$ (A-E) by Fisher's exact test (two-sided). (F-J) the number of viral particles in individual mosquito midguts (scatter plot) and mean (bar) are shown (Table S2); *$0.01\leq p<0.05$ ($p=0.0132$ for F, $p=0.0232$ for G), *** $0.0001\leq p<0.001$ ($p=0.001$ for H-J) by Kruskall-Wallis non-parametric test (one-sided). In brackets are the numbers of mosquitoes tested. Source data are provided as a Source Data file.

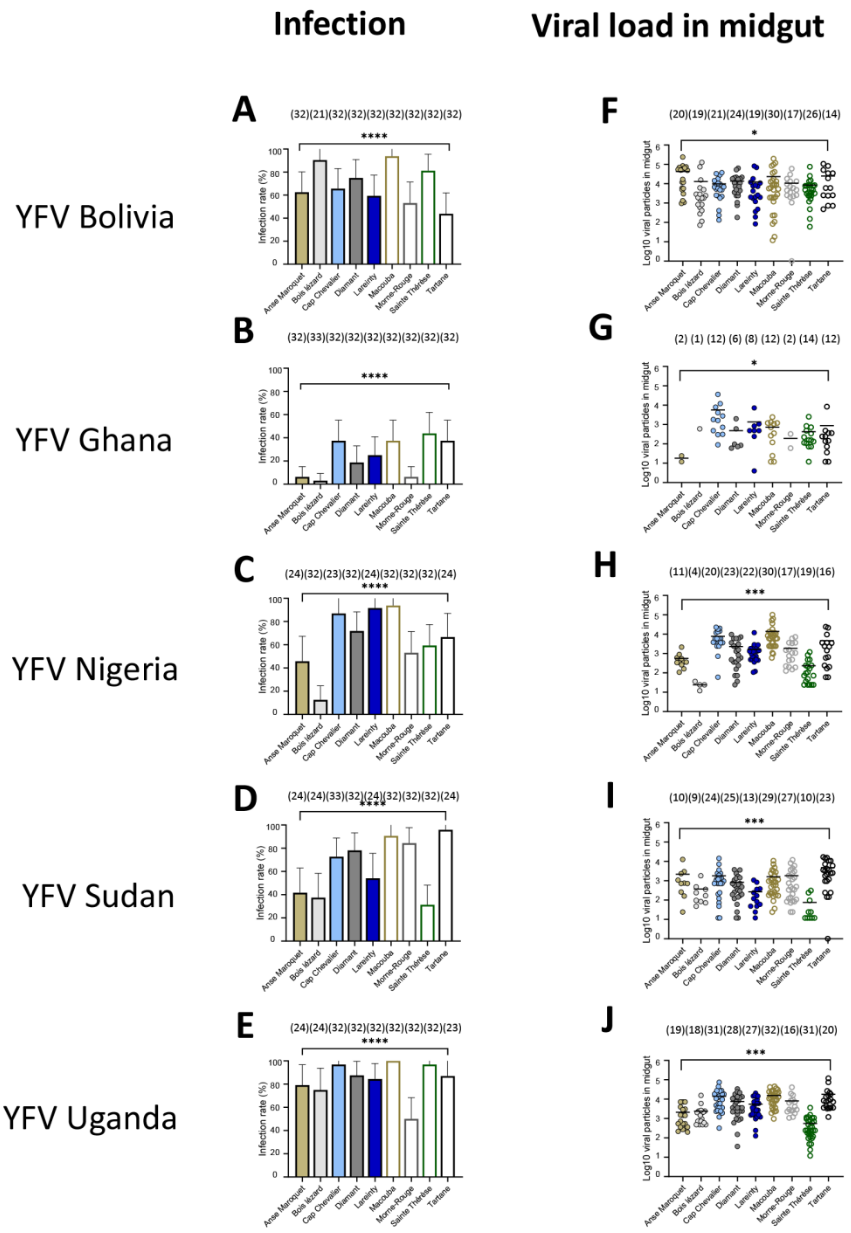

Figure S5. Stepwise dissemination rates (A-E) and viral loads in mosquito carcass (F-J) of nine *Aedes aegypti* populations from Martinique examined 14 days after exposure to an infectious blood meal containing one YFV strain (Bolivia, Ghana, Nigeria, Sudan or Uganda). Mosquitoes were exposed to an infectious blood meal at a titer of 10^7 FFU/mL using an Hemotek system maintained at 37 °C. Engorged mosquitoes were kept for 14 days in controlled conditions and then dissected to isolate the carcass for estimating the viral load by titration. (A-E) the stepwise dissemination rate was defined as the proportion of mosquitoes with an infected carcass among mosquitoes with an infected midgut. The error bars correspond to the confidence intervals (95%) for SDR (Table S2); ** 0.001≤p<0.01 (p=0.001 for D), ****p<0.0001 for A, C, E) by Fisher's exact test (two-sided). (F-J) the number of viral particles in individual carcasses (scatter plot) and mean (bar) are shown (Table S2); ** 0.001≤p<0.01 (p=0.0013 for H, p=0.022 for I), *** 0.0001≤p<0.001 (p=0.0002 for F, p=0.0001 for I) by Kruskall-Wallis non-parametric test (one-sided). In brackets are the numbers of mosquitoes tested. Source data are provided as a Source Data file.

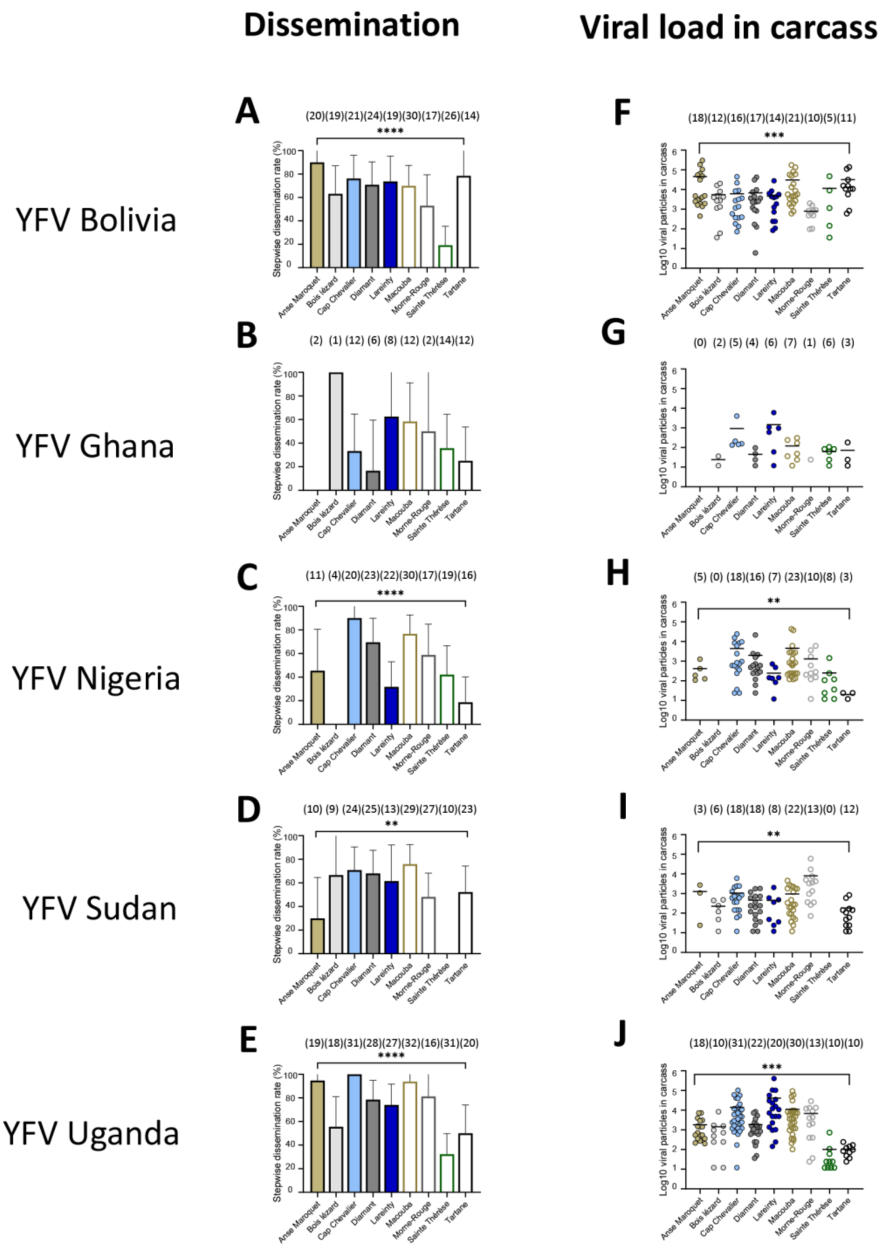

Figure S6. Stepwise transmission rates (A-E) and viral loads in mosquito saliva (F-J) of nine *Aedes aegypti* populations from Martinique examined 14 days after exposure to an infectious blood meal containing one YFV strain (Bolivia, Ghana, Nigeria, Sudan or Uganda). Mosquitoes were exposed an infectious blood meal at a titer of 10^7 FFU/mL using an Hemotek system maintained at 37 °C. Engorged mosquitoes were kept for 14 days in controlled conditions and then prepared for saliva collection to estimate the viral load by titration. (A-E) the stepwise transmission rate was defined as the proportion of mosquitoes with infectious saliva among mosquitoes with an infected carcass. The error bars correspond to the confidence intervals (95%) for STR (Table S2); *$0.01 \leq p < 0.05$ ($p=0.016$ for D), ** $0.001 \leq p < 0.01$ ($p=0.005$ for E) by Fisher's exact test (two-sided). (F-J) the number of viral particles in individual saliva (scatter plot) and mean (bar) are shown (Table S2). In brackets are the numbers of mosquitoes tested. Source data are provided as a Source Data file.

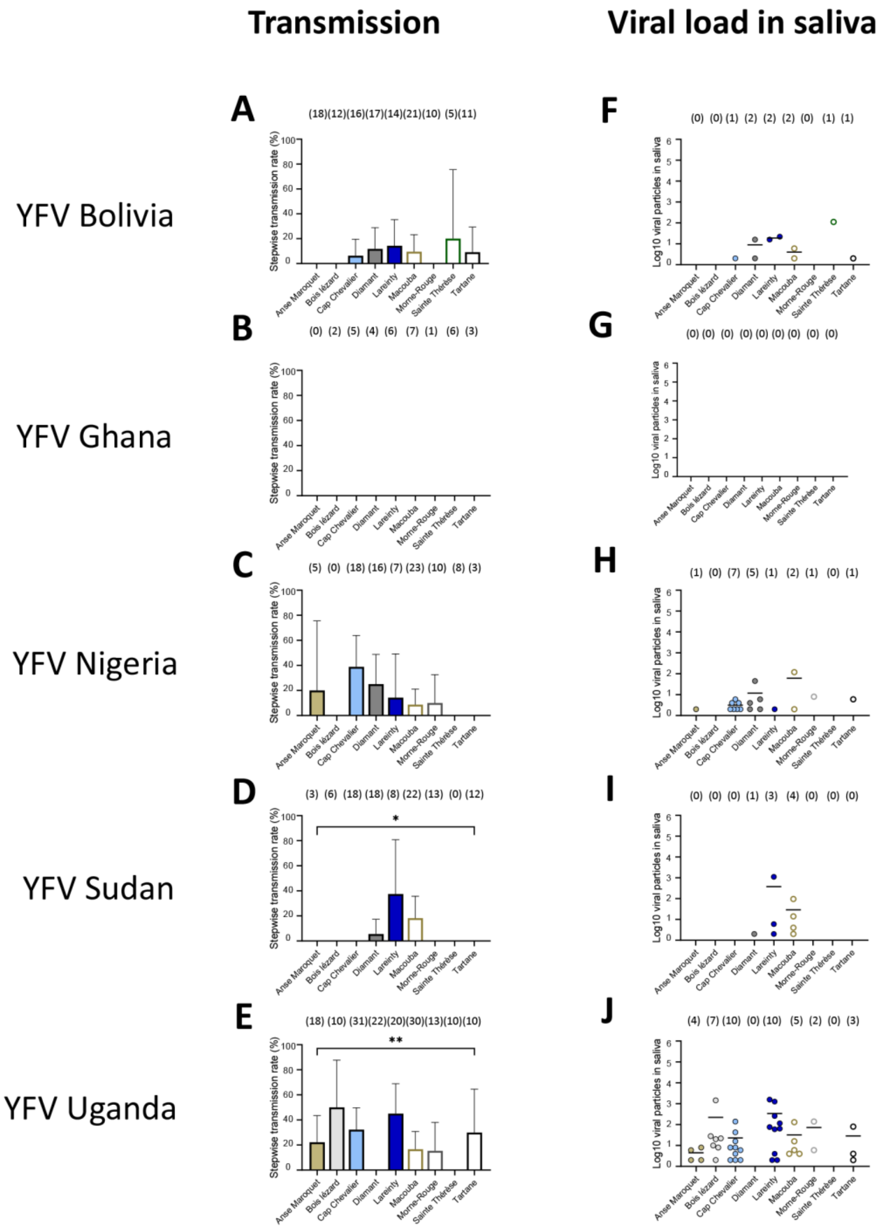

Figure S7. Number of virus particles (in Log_{10}) detected in the midgut of nine *Aedes aegypti* populations from Martinique according to mosquito status: with/without dissemination (A-E) and with/without transmission (F-J). Mosquitoes were examined 14 days after an infectious meal containing one YFV strain (Bolivia, Ghana, Nigeria, Sudan, and Uganda) provided at 10^7 FFU/mL. (A-E) the number of viral particles in individual midguts (scatter plot) and mean (bar) were estimated for mosquitoes able to disseminate the virus (detection of virus in carcass) *versus* mosquitoes unable to disseminate the virus (no detection of virus in carcass); *** 0.0001≤p<0.001 (p=0.0001 for A-E) by Kruskall-Wallis non-parametric test (one-sided). (F-J) the number of viral particles in individual midguts and mean (bar) were estimated for mosquitoes able to transmit the virus (detection of virus in saliva) *versus* mosquitoes unable to transmit the virus (no detection of virus in saliva); ***p<0.001 (p=0.0001 for H, p=0.0005 for J) by Kruskall-Wallis non-parametric test (one-sided). In brackets are the numbers of mosquitoes tested. Source data are provided as a Source Data file.

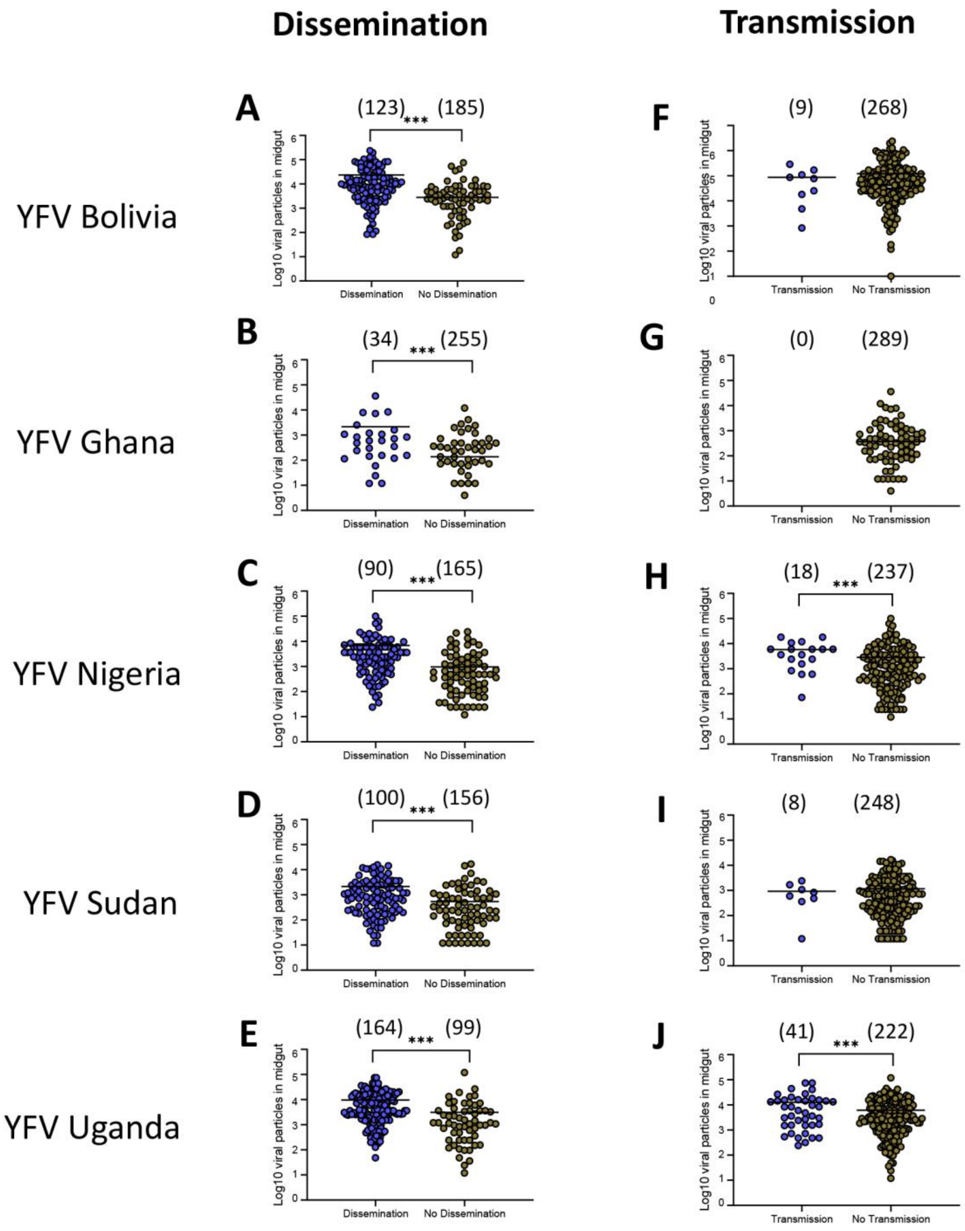

Figure S8. Number of virus particles (in Log_{10}) detected in the carcass of nine *Aedes aegypti* populations from Martinique according to mosquito status: with or without transmission. Mosquitoes were examined 14 (A-E) and 21 days (F-J) after an infectious meal containing one YFV strain (Bolivia, Ghana, Nigeria, Sudan, and Uganda) provided at 10^7 FFU/mL. The number of viral particles in individual mosquito carcasses (scatter plot) and mean (bar) were estimated for mosquitoes able to transmit the virus (detection of virus in saliva) *versus* mosquitoes unable to transmit the virus (no detection of virus in saliva). (A-E) **$0.001 \leq p \leq 0.01$ ($p=0.019$ for D), *** $0.0001 \leq p < 0.001$ ($p=0.0001$ for A, C, E) by Kruskall-Wallis non-parametric test (one-sided). (F-J) *$0.01 \leq p < 0.05$ ($p=0.0219$ for G), *** $0.0001 \leq p < 0.001$ ($p=0.0001$ for F, H-J) by Kruskall-Wallis non-parametric test (one-sided). In brackets are the numbers of mosquitoes tested. Source data are provided as a Source Data file.

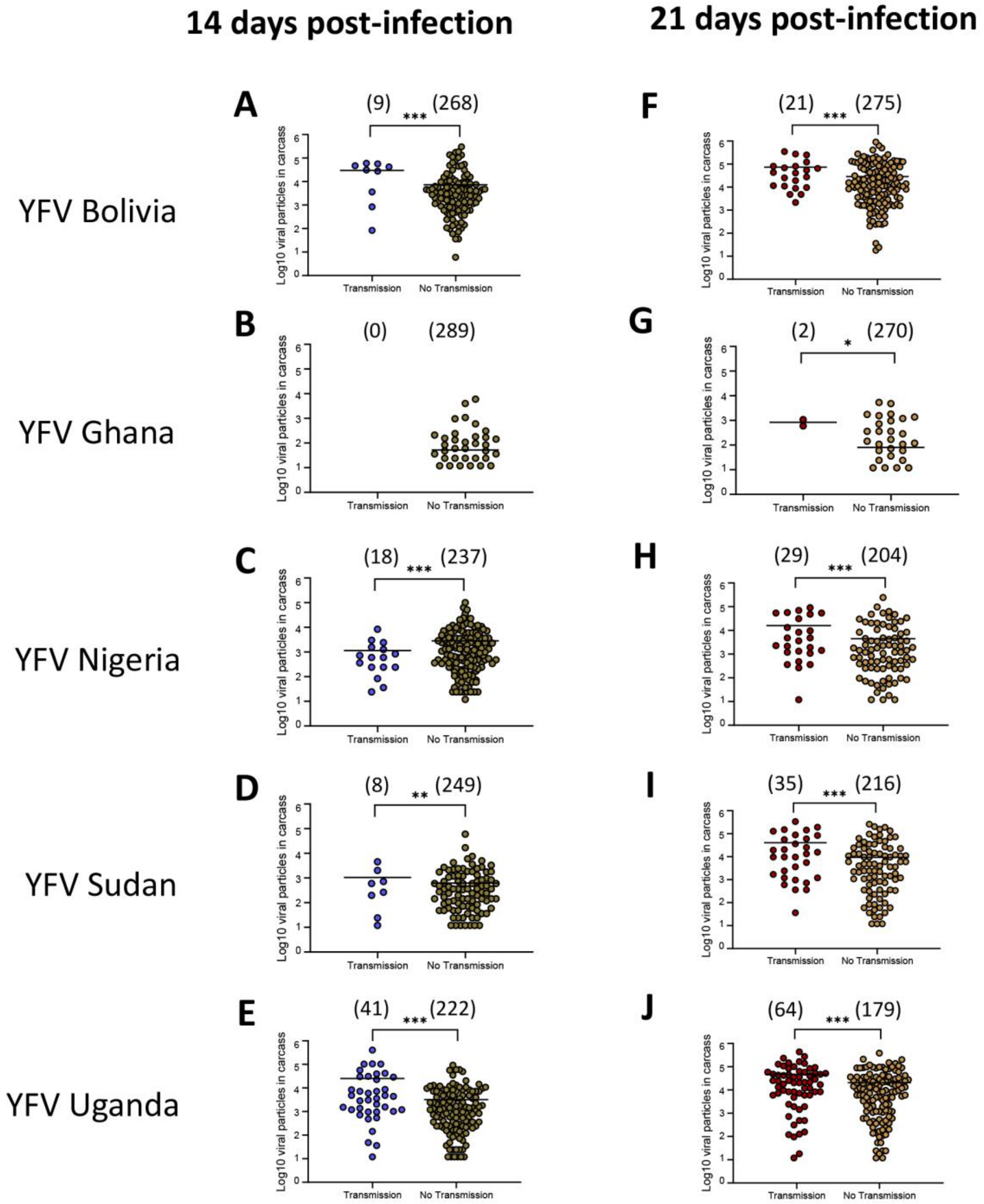

Figure S9. Model-predicted medians of cumulative transmission rates with 95%-credibility interval for all combinations of mosquito populations and YFV strain pairings examined at 14 and 21 days after exposure to an infectious blood meal containing one YFV strain (Bolivia, Ghana, Nigeria, Sudan or Uganda). Mosquitoes were exposed an infectious blood meal at a titer of 10^7 FFU/mL using an Hemotek system maintained at 37 °C. Engorged mosquitoes were kept for 14 and 21 days in controlled conditions and then prepared for saliva collection to estimate the viral load by titration. CTR refers to the proportion of mosquitoes with infectious saliva among all mosquitoes analyzed (having disseminated or not disseminated the virus). Source data are provided as a Source Data file.

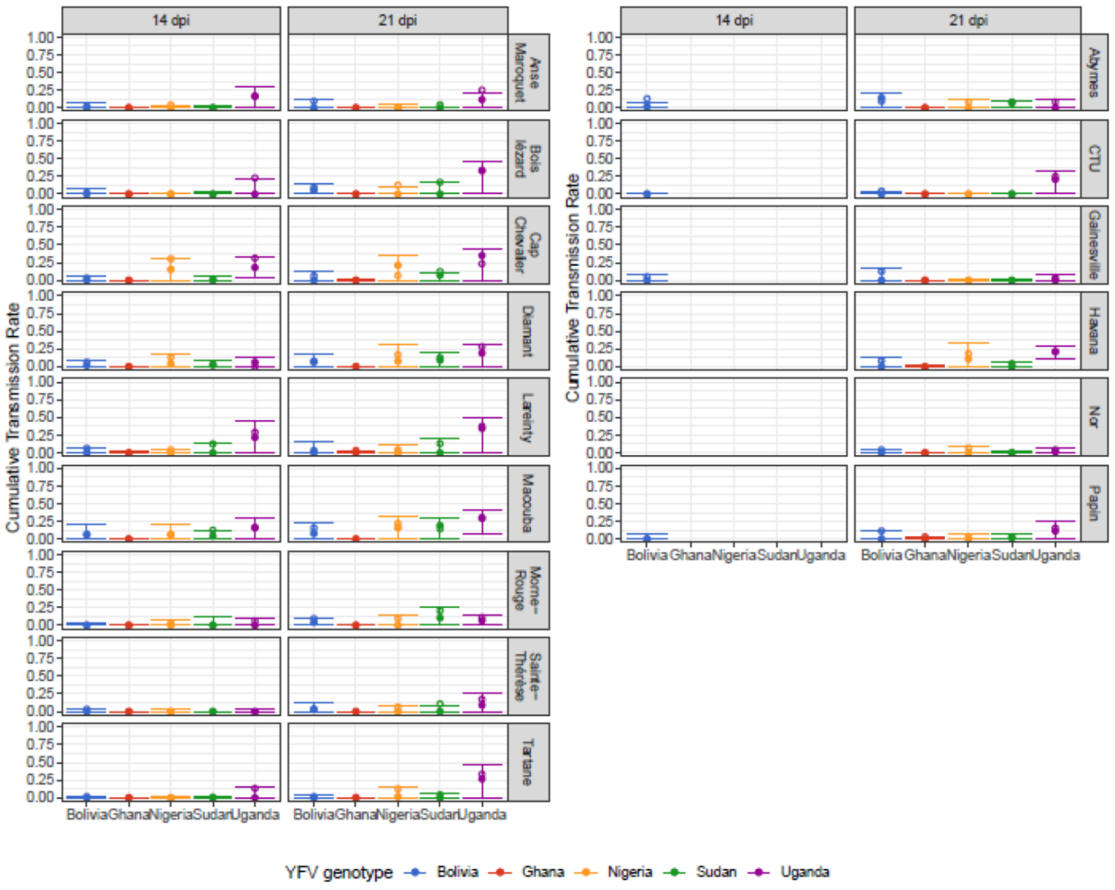

Supplement: Supplementary file 1 — Supplementary Information [file 41467_2024_45116_MOESM1_ESM.pdf]
